# The acceleration of reproductive aging in *Nrg1* ^*flox/flox*^ *;Cyp19‐Cre* female mice

**DOI:** 10.1111/acel.12662

**Published:** 2017-08-31

**Authors:** Takashi Umehara, Tomoko Kawai, Ikko Kawashima, Katsuhiro Tanaka, Satoshi Okuda, Hiroya Kitasaka, JoAnne S. Richards, Masayuki Shimada

**Affiliations:** ^1^ Graduate School of Biosphere Science Hiroshima University Higashi‐Hiroshima Japan; ^2^ Department of Molecular & Cellular Biology Baylor College of Medicine Houston TX USA

**Keywords:** estrus cycle, female fertility, gonadotropins, ovarian aging, ovarian stroma, steroidogenesis

## Abstract

Irregular menstrual cycles, reduced responses to exogenous hormonal treatments, and altered endocrine profiles (high FSH/high LH/low AMH) are observed in women with increasing age before menopause. In this study, because the granulosa cell‐specific *Nrg1* knockout mice (gc*Nrg1*KO) presented ovarian and endocrine phenotypes similar to older women, we sought to understand the mechanisms of ovarian aging and to develop a new strategy for improving fertility in older women prior to menopause. In the ovary of 6‐month‐old gc*Nrg1*KO mice, follicular development was blocked in bilayer secondary follicles and heterogeneous cells accumulated in ovarian stroma. The heterogeneous cells in ovarian stroma were distinguished as two different types: (i) the LH receptor‐positive endocrine cells and (ii) actin‐rich fibrotic cells expressing collagen. Both the endocrine and fibrotic cells disappeared following long‐term treatment with a GnRH antagonist, indicating that the high levels of serum LH induced the survival of both cell types and the abnormal endocrine profile to reduce fertility. Moreover, follicular development to the antral stages was observed with reduced LH and the disappearance of the abnormal stromal cells. Mice treated with the GnRH antagonist regained normal, recurrent estrous cycles and continuously delivered pups for at least for 3 months. We conclude that endocrine and matrix alternations occur within the ovarian stroma with increasing age and that abolishing these alternations resets the cyclical release of LH. Thus, GnRH antagonist treatments might provide a new, noninvasive strategy for improving fertility in a subset of aging women before menopause.

## Introduction

Female fertility declines with increasing age not only in humans but also in other species including mice and livestock animals. Fertility of women decreases slightly around the age of 30 (van Noord‐Zaadstra *et al*., 1991), but is clearly evident at a median age of 40 (Bongaarts, 1982). Several reports indicate that the decline in fertility with age is associated with the loss of follicles and the poor quality of oocytes in these follicles (Gosden *et al*., 1983; Faddy *et al*., 1992). However, there are about 10 000 follicles in ovary of women over 40 years of age (Faddy *et al*., 1992) and woman beyond 40 years can get pregnant following hormone treatments used in assisted reproduction technology (ART) (Manson & Martin, 2001; Klipstein *et al*., 2005). These observations indicate that the decline in fertility in women over 40 years of age until menopause is related not only to the loss of follicles in ovary but also to either abnormal hormone levels or abnormal responses to hormones in the hypothalamic–pituitary–gonadal axis.

In women, menstrual cycles become irregular at about 40 years of age (Treloar, 1981), although it takes another 10 years until menstrual cycles cease (den Tonkelaar *et al*., 1998). Abnormal estrous cycles are also observed with increasing age in mice (Nelson *et al*., 1981), rats (Fugo & Butcher, 1971), and cattle (Malhi *et al*., 2005). Malhi *et al*. (2005) reported that the estrous cycle is prolonged due to altered periodic changes of estradiol and FSH, which results in the extinction of normal estrous cycles (Malhi *et al*., 2005). Beginning around 10 months of age, female mice also exhibit an extended estrous phase (Nelson *et al*., 1981) and a poor response to standard super‐ovulatory regimens of FSH/eCG and LH/hCG; the number of ovulated oocytes is significantly lower than that in younger mice (Holinka *et al*., 1979; Mori, 1979). The ability of follicles to respond to both gonadotropins is acquired at the secondary stage when follicles have multiple layers of granulosa cells (Dierich *et al*., 1998), suggesting that follicular development beyond the secondary stage is suppressed, resulting perhaps in the accumulation of secondary follicles in the ovary of aged mice and women. The development from secondary to antral follicles is dependent on the basal levels of serum FSH and LH that are regulated by hypothalamic release of GnRH as well as by ovarian factors, such as inhibin B, estradiol, and progesterone (Ling *et al*., 1987; Couse & Korach, 1999).

Based on these considerations, we hypothesized that irregular secretion of gonadotropins and altered ovarian feedback mechanisms might impact ovarian follicular development in aged female mice leading to abnormal estrous cycles. Unfortunately, there is limited information about the relationship of the ovarian–hypothalamic–pituitary axis and follicular development in aging female mice. Most studies focus on the decline of female fertility and the loss of follicles/oocytes in ovary; only a few reports mention the process by which ovarian functions change with increasing age.

Anti‐Mullerian hormone (AMH) is expressed at high levels in pre‐antral follicles and, based on the decrease in AMH in serum of aging women, it is used as an indicator of the follicle reserve. However, AMH levels are not different in mice between 10 and 14 months of age and mice that are younger (Kevenaar *et al*., 2006). Therefore, it is thought that wild‐type mice are not a relevant model to understand the mechanisms by which ovarian function declines with increasing age in older women. In our previous studies, the expression of neuregulin 1 (NRG1) was observed in LH‐stimulated granulosa cells during ovulation. In the granulosa cell‐specific *Nrg1* KO mice (gc*Nrg1*KO), rupture of the follicular wall occurs; however, not only the number of pups delivered declined in the mutant mice but also the time interval between litters was prolonged in the mutant mice more than 6 months of age (Kawashima *et al*., 2014). Thus, we used this mutant mouse model to determine the underlying causes of the prolonged intervals between litters in these mice older than 6 months age and to examine whether the mechanisms occurring in the mutant mice might also apply to altered fertility in aged wild‐type (WT) mice. Finally, we developed a method to restore the normal ovarian function and fertility in aged mice.

## Results

### The decline of fertility with increasing age is accelerated in gc*Nrg1*KO mice

**Figure 1:**
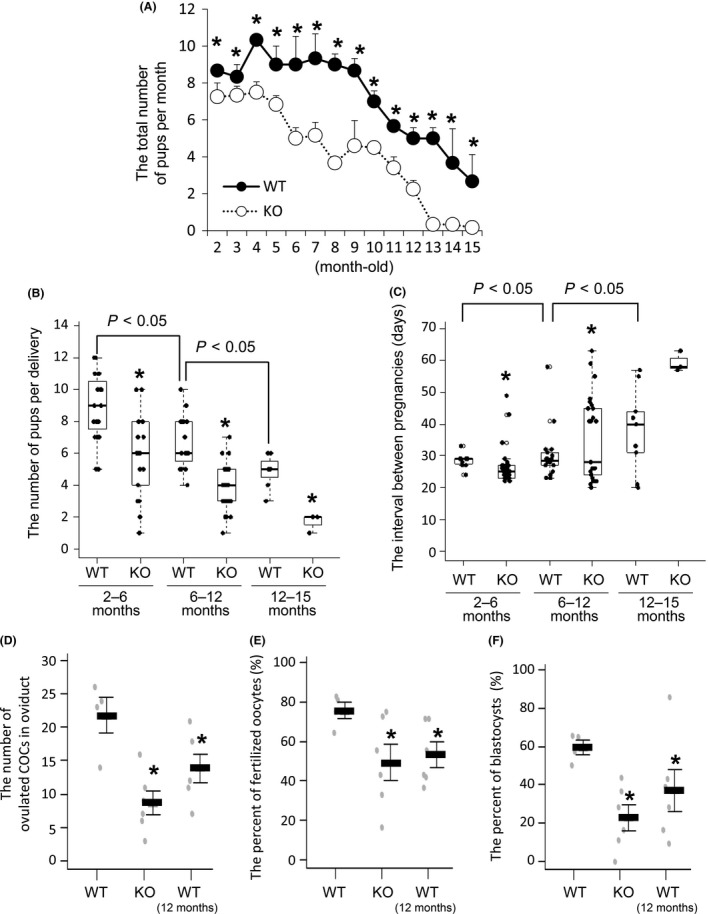
The decline of fertility with increasing age is accelerated in gc*Nrg1*KO mice. (A) The total number of pups born per month in each gc*Nrg1KO* (KO) female and WT male mating pair or each *Nrg1* ^*flox/flox*^ (WT) female and WT male mating pair were determined during a 15‐month breeding period. Five pairs were analyzed in each genotype. *denotes a significant difference between genotypes (*P* < 0.05). (B) The average number of pups per litter was calculated in each gc*Nrg1KO* (KO) female and WT male mating pair or each *Nrg1* ^*flox/flox*^ (WT) female and WT male mating pairs. Five pairs were prepared in each genotype. *denotes a significant difference between genotypes (*P* < 0.05). In the box plot: bar = median, box = 25th to 75th percentiles, whiskers = 10th and 90th percentile. (C) The intervals between pregnancies were calculated in each gc*Nrg1KO* (KO) female and WT male mating pair or each *Nrg1* ^*flox/flox*^ (WT) female and WT male mating pair. Five pairs were prepared in each genotype. The data were statistically analyzed by Bartlett's test. *denotes a significant difference between genotypes (*P* < 0.05). In the box plot: bar = median, box = 25th to 75th percentiles, whiskers = 10th and 90th percentile. (D, E, F) The number of ovulated oocytes (D), the percent of fertilization oocytes per ovulated oocytes (E), and the percent of blastocysts per fertilization oocytes (F) in 6‐month‐old WT, 6‐month‐old gc*Nrg1*KO, and 12‐month‐old WT. Each mouse at diestrus was injected ip with 4 IU eCG followed by 48 h later with 5 IU of hCG. After 16 h, the ovulated COCs were collected from oviducts and then were subjected to *in vitro* fertilization. Black bars indicated mean value of each percent. *, *P* < 0.05, significant differences were observed compared with 6‐month‐old WT.

In WT mice, the number of pups born per month decreased linearly from 9 to 15 months of age (Fig. 1A). The number of pups delivered from each pregnancy also decreased significantly with age (Fig. 1B). The interval between pregnancies was about 30 days; however, the interval increased after 12 months of age with large variations (Fig. 1C). In the granulosa cell‐specific *Nrg1*KO (gc*Nrg1*KO) mice, the number of pups born per month was significantly lower than that in WT mice during all mating periods with linear decreases observed from 6 to 8 and then 10 to 13 months of age after which very few pups were born (Fig. 1A). Reduced fertility in the gc*Nrg1*KO mice was associated with both the longer interval between pregnancies and the decreased number of pups delivered (Fig. 1A–C). Specifically, the interval between pregnancies was highly variable in gc*Nrg1*KO mice and exceeded more than 50 days after 12 months of age (Fig. 1C). When mice were injected with super‐ovulatory doses of eCG followed 48 h later with hCG, the number of ovulated COCs in oviduct of 6‐month‐old gc*Nrg1*KO mice was lower compared with that in 6‐month‐old WT mice (Fig. 1D). A low number of ovulated oocytes were also detected in 12‐month‐old WT mice (Fig. 1D). Fertilization rates of oocytes and the competence of these fertilized oocytes to develop to the blastocyst stage were significantly lower in oocytes recovered from 6‐month‐old gc*Nrg1*KO or 12‐month‐old WT mice than those in 6‐month‐old WT, respectively (Fig. 1E,F).

### Abnormal estrous cycles occur with increasing age

**Figure 2:**
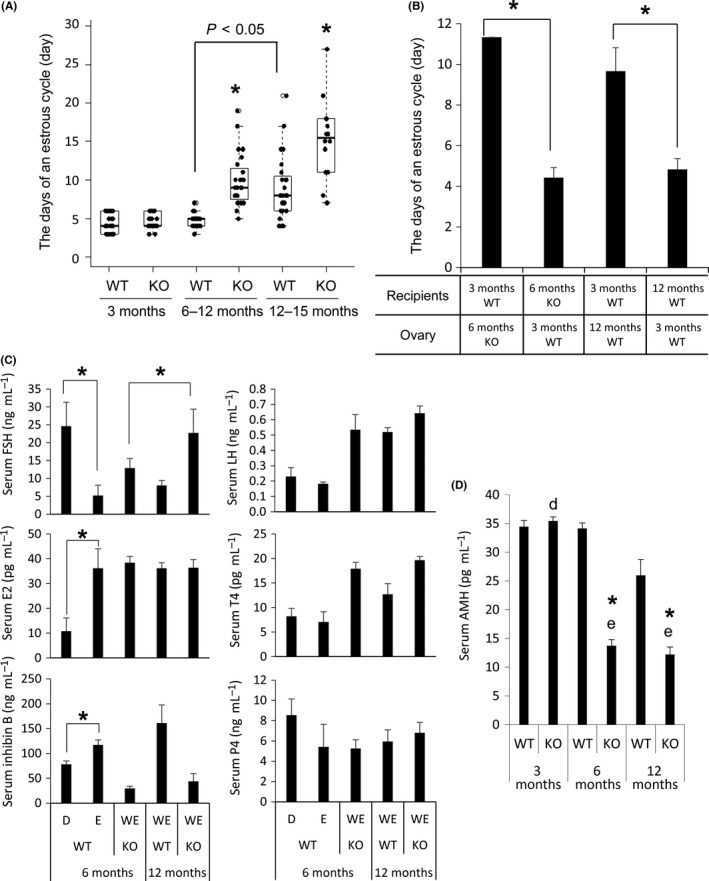
The abnormal estrous cycle is induced by the changes in the ovary that occur with increasing age. (A) Estrous cycle length (in days) in WT and gc*Nrg1*KO mice 3–12 months old . Smears were obtained daily between 09:00 and 10:00 am. The fire‐polished, shortened tip of a Pasteur pipet was placed at the vagina. *denotes a significant difference (*P* < 0.05). In the box plot: bar = median, box = 25th to 75th percentiles, whiskers = 10th and 90th percentile. (B) Estrous cycle length (in days) in the mice following transplantation of ovaries between 6‐month‐old WT and gc*Nrg1KO* or between 6‐month‐old WT and aged WT (12‐month‐old). *denotes a significant difference (*P* < 0.05). (C) Serum levels of FSH, LH, estradiol (E2), testosterone (T4), inhibin B, and progesterone (P4) in WT and gc*Nrg1*KO. The samples of 6‐month‐old WT were collected at diestrus and estrus. In 6‐month‐old gc*Nrg1*KO mice and 12‐month‐old mice of both genotypes, the samples were collected in weak estrus because other estrus periods were rarely observed. The levels of each hormone were measured by EIA kit. *N* = 4 animals for each genotype. Values are represented as the mean ± SEM of four replicates. *denotes the significant differences observed (*P* < 0.05). (D) Serum levels of AMH in each age of WT and gc*Nrg1*KO mice. The samples were collected 2 days after estrus, and AMH levels were measured by EIA kit. *N* = 4 animals for each genotype. Values are represented as the mean ± SEM of four replicates. *denotes the significant differences observed between genotypes at the same age (*P* < 0.05). Different superscripts denote significant differences among the age in each genotype (*P* < 0.05).

Estrous cycles of 4 or 5 days occurred in 6‐month‐old WT mice (Figs 2A and S1, Supporting information). However, the estrous cycles in gc*Nrg1*KO mice beyond 6 months of age and WT mice beyond 12 months of age were abnormal. Rascop *et al*. (1966) defined ‘weak estrus’ (WE) when not only many cornified epithelial cells but also leukocytes were observed in the vaginal smear; hence, gc*Nrg1*KO mice beyond 6 months and WT mice beyond 12 months presented weak estrus (Fig. S1, Supporting information). In addition, the estrous cycle of gc*Nrg1*KO over 6 months or WT mice older than 12 months, respectively, was significantly longer than that in 6‐ to 12‐month‐old WT mice (Fig. 2A).

To determine the underlying causes of the onset of abnormal estrous cycle length of gc*Nrg1*KO mice, ovarian transplantation experiments were carried out. When ovaries of 6‐month‐old gc*Nrg1*KO mice were transplanted under the ovarian bursa of 3‐month‐old WT mice, the estrous cycle was prolonged and similar in length to that observed in the gc*Nrg1*KO mice. Conversely, when ovaries of WT mice were transplanted into the mutant mice, normal estrous cycle length was observed, indicating that the WT ovary restored normal cycle length in the mutant mice. The estrous cycle in 12‐month‐old WT mice was also normalized by the transplantation of ovaries from 3‐month‐old WT mice (Fig. 2B), indicating that the irregular estrous cycles caused by the abnormal functions of *Nrg1*‐mutant ovaries and by the alternation of ovarian function with increasing age.

In 6‐month‐old WT mice, serum levels of FSH decreased from diestrus to estrus and this was associated with increased levels of inhibin B and estradiol 17β (E2), a pattern observed in mice with normal fertility (Figs 2C and S2, Supporting information). However, gc*Nrg1*KO mice showed a stable endocrine condition, elevated levels of FSH and E2, and lower levels of inhibin B on the day of weak estrus. Serum levels of FSH, LH, E2, and testosterone (T4) were higher than those of their basal levels in 6‐month‐old WT mice. The level of FSH was further increased in 12‐month‐old gc*Nrg1*KO as compared with that of gc*Nrg1*KO mice at 6 months (Figs 2C and S2, Supporting information). No significant difference of serum levels of AMH level was observed between WT and mutant mice at 3 months of age. However, serum levels of AMH were significantly lower in 6‐month‐old or 12‐month‐old gc*Nrg1*KO mice as compared with that in same age of WT mice, respectively (Fig. 2D).

### Ovarian morphology changes with increasing age and is accelerated in gc*Nrg1*KO mice

**Figure 3:**
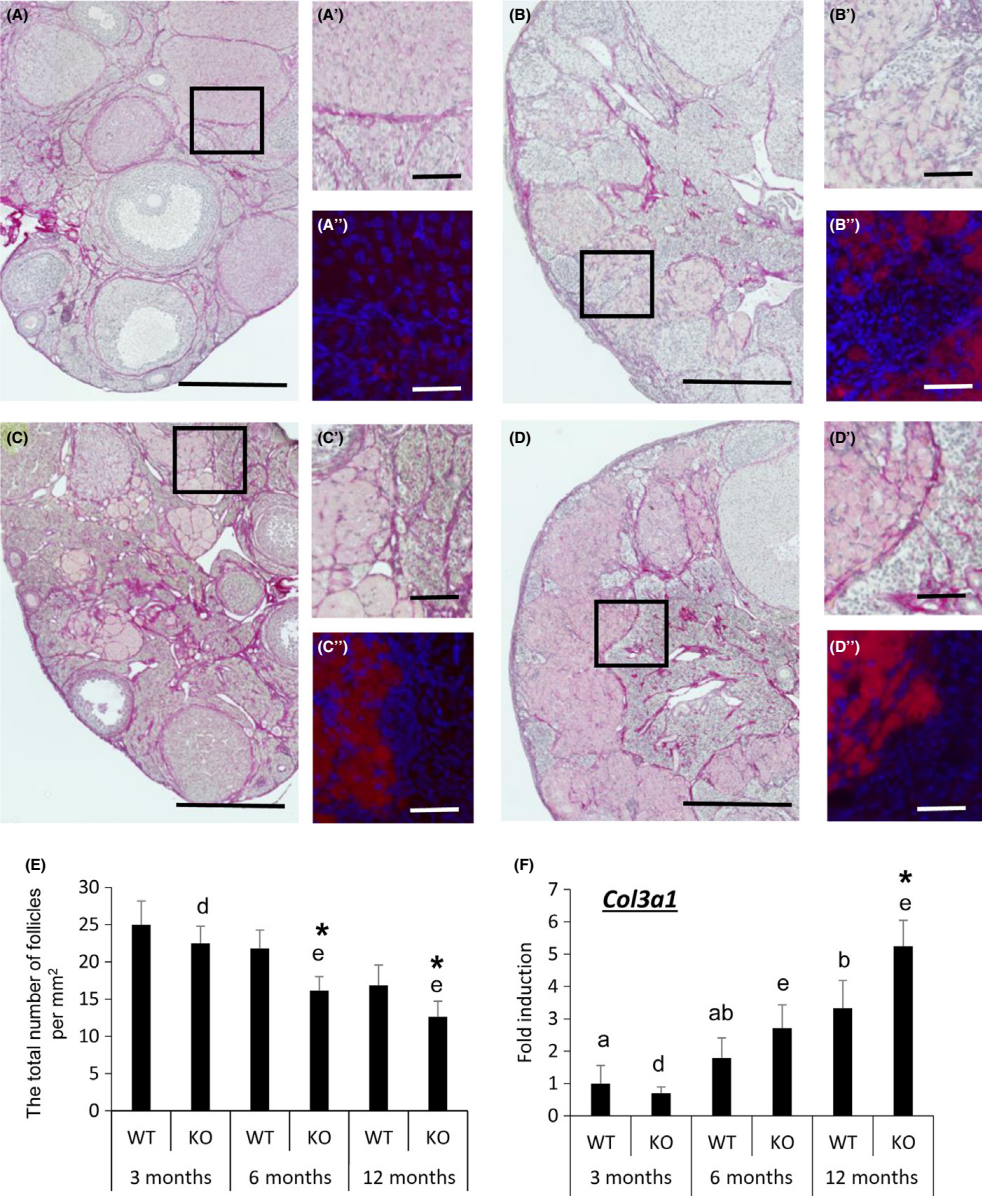
Changes in ovarian morphology are accelerated with increasing age in the gc*Nrg1*KO mice. (A,B,C,D) Picrosirius red (PSR) staining of 6‐month‐old WT (A), 6‐month‐old gc*Nrg1KO* (B), 12‐month‐old WT (C), and 12‐month‐old gc*Nrg1KO* (D) ovaries. Ovaries collected 2 days after estrus were fixed by 4 % paraformaldehyde. The sections were stained by picrosirius red and eosin. The scale bar for images in (A–D) is 400 μm. (A′–D′ and A″–D″) are higher magnification images from specific regions of the ovarian stroma that are boxed in (A–D). (A″–D″) are fluorescence images using rhodamine phalloidin that recognizes F‐actin and DAPI that recognizes nuclei, with constant settings that were established for the 12‐month‐old gc*Nrg1*KO (D″). Scale bars in (A′–D′ and A″–D″) are 100 μm. (E) The total number of follicles per mm^2^ in ovaries of WT and gc*Nrg1*KO mice at each age (*n* = 3 ovaries per each group). Sections taken at intervals of 30 μm and 6 μm of paraffin‐embedded ovaries were mounted on slide. Follicle numbers in 12 sections per ovary were evaluated. Values are represented as the mean ± SEM of three replicates. *denotes the significant differences observed between genotypes at the same age (*P* < 0.05). Different superscripts denote significant differences among the age in each genotype (*P* < 0.05). (F) The expression of *Col3a1* in ovaries of WT and gc*Nrg1*KO mice. The ovaries were collected 2 days after estrus. The expression levels of genes were normalized according to that of *L19*. Values represent mean ± SEM of 3 replicates. The data were statistically analyzed by a two‐way ANOVA. If a significant difference was observed in each factor, the comparison analysis was performed by a Student's *t*‐test. Different superscripts denote significant differences among the age in each genotype (*P* < 0.05). *denotes significant differences observed in each age between genotypes.

In the ovary of 6‐month‐old WT mice, many antral follicles and corpora lutea were observed (Fig. 3A), and picrosirius red (PSR) staining of collagen (I and III) fibers was intense in the basal lamina of growing follicles and in the stroma; only weak signals were detected in corpora lutea (Fig. 3A′). In the ovary of 6‐month‐old gc*Nrg1*KO mice, large round cells in the stroma were stained by PSR (Fig. 3B′, D’). The PSR‐positive cells were also rhodamine phalloidin positive, a marker of F‐actin (Fig. 3B″,D″, Fig. S3, supporting information). PSR and rhodamine phalloidin staining increased in the stroma of 12‐month‐old mutant mice (Fig. S3, supporting information). In the ovary of 6‐ and 12‐month‐old gc*Nrg1*KO mice, fewer secondary follicles and antral follicles were observed; however, the number of bilayer stage secondary follicles was significantly higher than that in ovaries of WT mice of the same age (Figs 3B,D and S4, Supporting information). In association with the increased number of PSR‐stained cells, the expression of *Col3a1* mRNA in ovaries of 12‐month‐old WT mice was significantly higher than that in ovaries of 3‐month‐old WT mice. The induction of *Col3a1* was accelerated in ovaries of the mutant mice; the expression in the ovary of 6‐month‐old gc*Nrg1*KO mice was significantly higher than that of 3‐month‐old gc*Nrg1*KO mice (Fig. 3F).

### Ovarian functions change with increasing age and are accelerated in gc*Nrg1*KO mice

**Figure 4:**
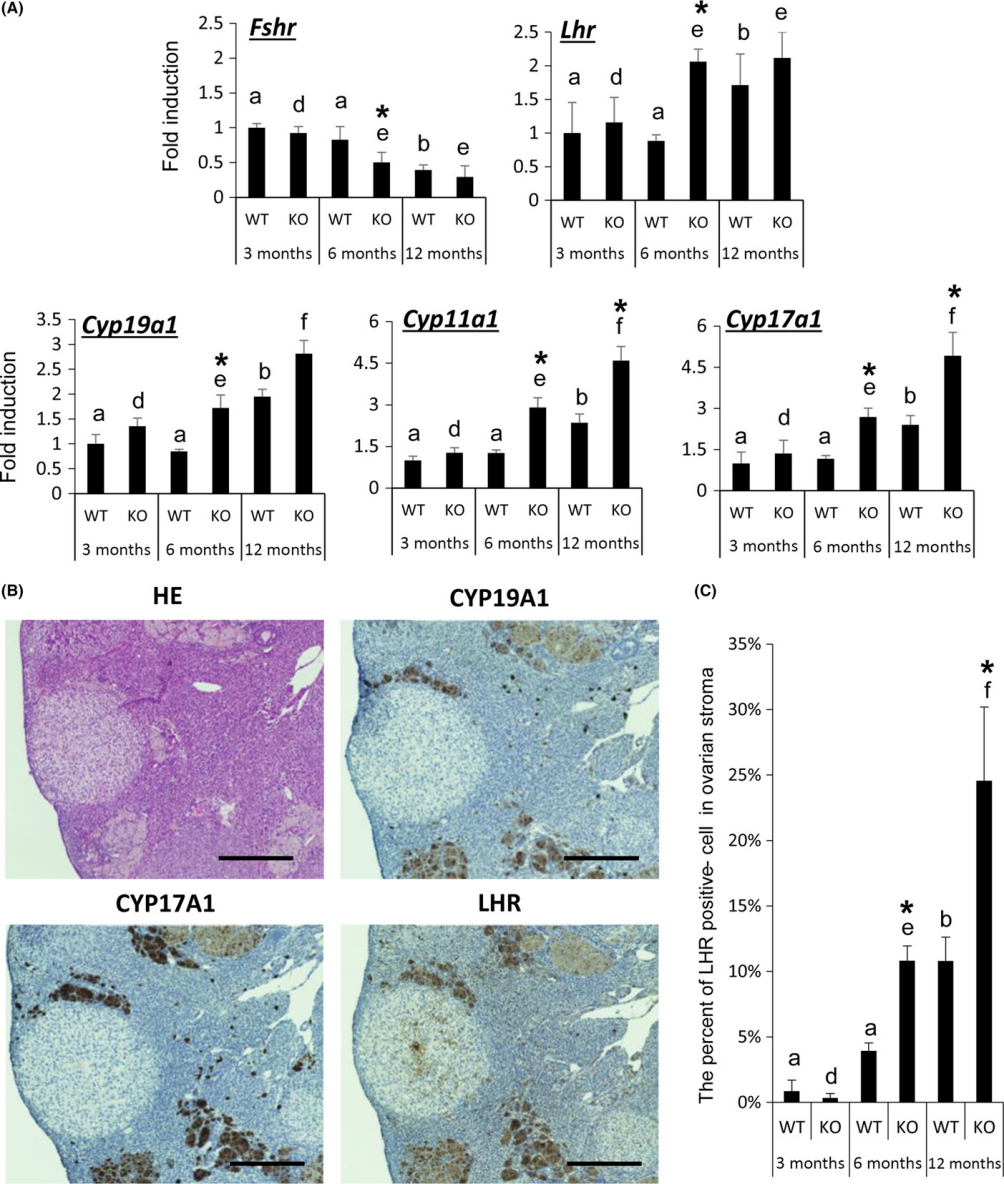
Heterogeneous cells that accumulate in ovarian stroma potentially have a steroidogenic activity. (A) The expression levels of *Fshr, Lhr, Cyp19a1, Cyp11a1,* and *Cyp17a1* in ovaries of WT or gc*Nrg1KO* (KO) mice. The ovaries were collected 2 days after estrus. The expression levels of genes were normalized according to that of *L19*. Values represent mean ± SEM of three replicates. The data were statistically analyzed by a two‐way ANOVA. If a significant difference was observed in each factor, the comparison analysis was performed by a Student's *t*‐test. Different superscripts denote significant differences among the age in each genotype (*P* < 0.05). *denotes significant differences observed in each age between genotypes. (B) The localization of the heterogeneous, CYP19‐positive, CYP17‐positive, and LHR‐positive cells in ovaries of 6‐month‐old gc*Nrg1KO* mice. Scale bars correspond to 300 μm (×100). (C) The percent of LHR‐positive cells in ovarian stroma of WT and *gcNrg1KO* (*n* = 3 in each group). Sections taken at intervals of 30 μm and 6 μm from paraffin‐embedded ovariess were mounted on slide. The sections were stained using anti‐LHR antibody, and the positive cells were cells counted. The data were statistically analyzed by a two‐way ANOVA. If the significant difference was observed in each factor, the comparison analysis was performed by a Student's *t*‐test. Different superscripts denote significant differences among the age in each genotype (*P* < 0.05). *denotes significant differences observed in each age between genotypes.

The expression of *Fshr* mRNA, a marker of multiple layers of secondary follicles and antral follicles, decreased in ovaries of WT mice with increasing age; decreased *Fshr* expression was accelerated in gc*Nrg1*KO and appears to reflect the early alteration of follicular development in these mutant mice (Fig. 4A). However, the expression of markers of secondary and antral follicles, *Lhcgr*,* Cyp11a1*,* Cyp17a1*, and *Cyp19a1*, was significantly higher in ovaries collected from mutant mice at 6 months of age as compared with expression in ovaries of younger mutant mice or same age WT mice. Significant increases in the expression of these genes were also observed in the ovaries of 12‐month‐old WT and gc*Nrg1*KO mice (Fig. 4A). To determine which cell type(s) expressed the markers in ovary, IHC studies were carried out using anti‐CYP19a1, anti‐CYP17a1, and anti‐LHCGR antibodies. The positive signals for anti‐CYP19a1 antibody and anti‐LHCGR antibody were observed in granulosa cells of pre‐ovulatory follicles in the ovary of eCG‐primed WT mice (Fig. S5, Supporting information). The signals for anti‐CYP17a1 antibody were localized in theca cells of antral follicle in the ovary of eCG‐primed WT mice (Fig. S5, Supporting information). However, in ovaries of the gc*Nrg1*KO mice, intense staining for these follicular markers was observed in cells within the ovarian stroma rather than within ovarian follicles (Fig. 4B). The percent of anti‐LHCGR‐positive cells in ovarian stroma of gc*Nrg1*KO mice was significantly higher than that in same age mice (Fig. 4C). The increase in anti‐LHCGR‐positive cells in ovarian stroma was observed ovaries of WT and mutant mice with increasing age (Fig. 4C).

### GnRH antagonist treatment restored normal functions of the ovary in 6‐month‐old gc*Nrg1*KO

The cells expressing LH receptor accumulated in the ovarian stroma with increasing age (Fig. 4B), and serum levels of LH and FSH in gc*Nrg1*KO mice were consistently higher than those in WT mice (Fig. 2C), indicating that the function and/or survival of these cells might be dependent on the gonadotropin stimulation. To analyze this hypothesis, we suppressed gonadotropin secretion from pituitary gland by GnRH antagonist treatment.

**Figure 5:**
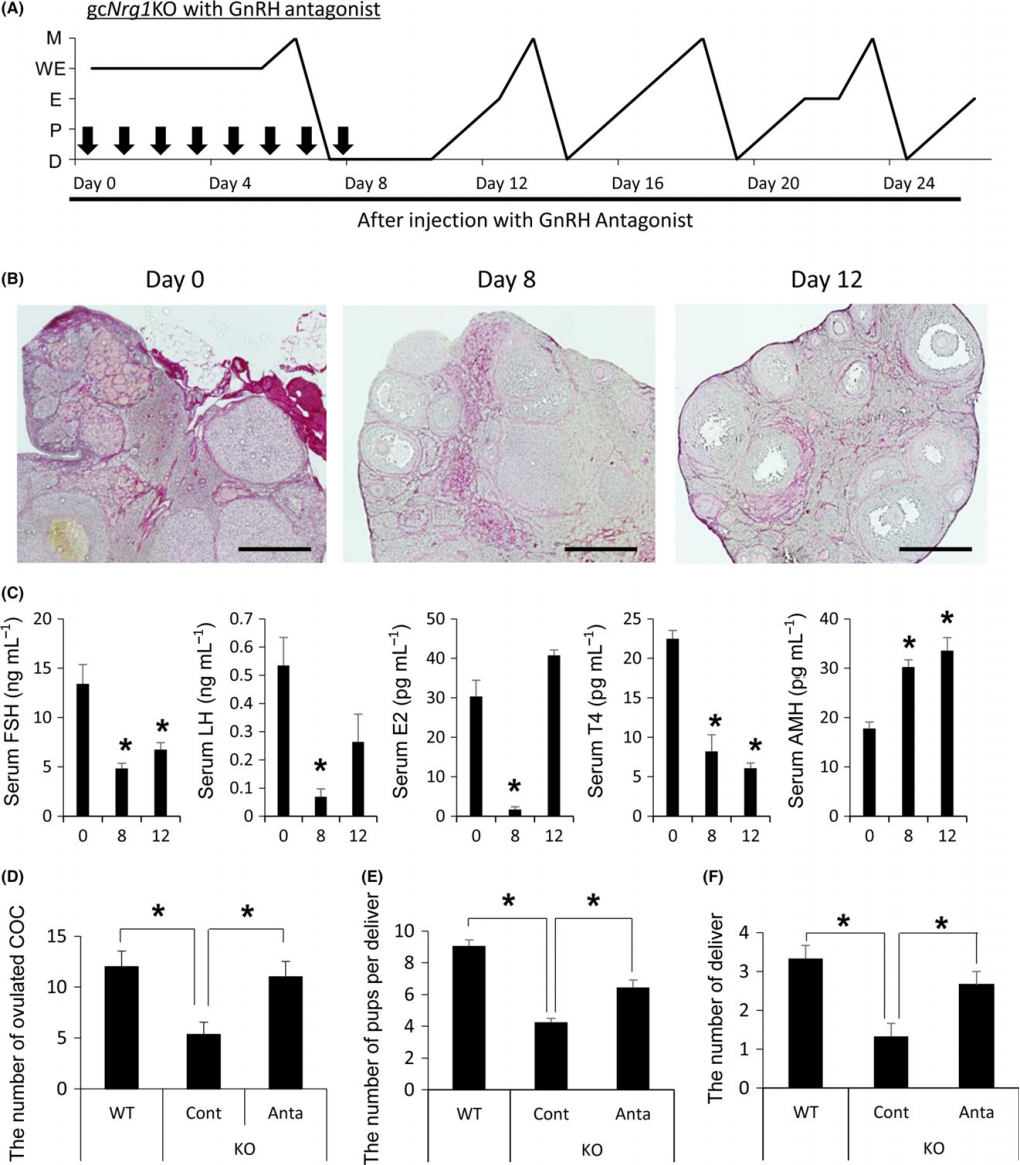
GnRH antagonist treatment induces apoptosis of heterogeneous cells in the ovarian stroma and restores ovarian functions in 6‐month‐old gc*Nrg1*KO mice. (A) The pattern of estrous cycles for 30 days of 6‐month‐old *gcNrg1KO* mice 7 days after GnRH antagonist treatment. Black allows show the time when the GnRH antagonist injection was stopped. (D, diestrus; P, Proestrus; E, Estrus; WE, Weak estrus; M, Metestrus) (B) Histological analysis of ovaries collected from day 0, day 8, and day 12. The ovaries were stained by picrosirius red and eosin. The scale bar for images in (A–F) is 400 μm. The images in the black square are higher magnification images. The scale bar for images is 100 μm. (C) Serum levels of FSH, estrogen, testosterone, and AMH after the start of GnRH antagonist treatment. *N* = 4 animals for each point. Values are represented as the mean ± SEM of four replicates. *, *P* < 0.05, significant differences were observed compared with Day 0. (D) The number of ovulated oocytes in 6‐month‐old WT, 6‐month‐old gc*Nrg1KO*, and 6‐month‐old gc*Nrg1KO* mice treated with GnRH antagonist. Mice at proestrus were injected ip with 5 I.U hCG. After 16 h, the ovulated COCs were collected from oviducts and the number of ovulated COCs was counted. (E) The number of pups per delivered was calculated for each gc*Nrg1KO* female mouse treated with saline (KO Cont) or GnRH antagonist (KO Anta) during mating with a WT male. Three pairs were analyzed in each treatment. *denotes a significant difference (*P* < 0.05). (F) The number of pups delivered during 3 months was calculated in each gc*Nrg1KO* female mouse treated with saline (KO Cont) or GnRH antagonist (KO Anta) during mating with a WT male. Three pairs were prepared in each treatment. *denotes a significant difference (*P* < 0.05).

Following 8 days of treatment with the GnRH antagonist, the mutant mice entered a normal estrous cycle, progressing from estrus to diestrus rather than from estrus to weak estrus (Fig. 5A). With the return of normal estrous cycles we observed, reduced PSR staining (indicative of decreased collagen) in the stroma, increased staining of anticleaved caspase 3 (indicative of increased apoptosis), reduced stromal cell staining of anti‐LHR (indicative of decreased LH‐target cells) and anti‐CYP19 (indicative of decreased estrogen production) (Figs 5B and S6A, Supporting information), and decreased serum levels of LH, FSH, estradiol, and testosterone (Fig. 5C). At day 8 after GnRH antagonist treatment, the percent of multilayered secondary follicles (Fig. S6B, Supporting information) and serum levels of AMH (Fig. 5C) were significantly increased as compared with those before the treatment.

The GnRH antagonist treatment was terminated after 8 days when all of the treated gc*Nrg1*KO mice exhibited a diestrous smear rather than a weak estrous smear. The first estrus was observed 5 days after the final GnRH antagonist injection, and estrus was observed every 4 or 5 days thereafter (Fig. 5A). Antral follicles were observed in the ovary of gc*Nrg1*KO at the end of GnRH antagonist treatment, and antral follicles developed to large antral follicles 4 days later (Fig. 5B). Injections of hCG on pro‐estrous increased the number of ovulated COCs in the oviducts of mutant mice as compared with that of age‐matched nontreated mutant mice (Fig. 5D). To determine the fertility of the gc*Nrg1*KO mice after the GnRH antagonist treatment, the mutant mice were mated with WT male mice for 3 months. The total number of pups delivered per pregnancy and the number of pregnancies increased following the GnRH antagonist treatment regimen (Fig. 5E,F). The extended GnRH antagonist treatment also normalized the estrous cycles of 12‐month‐old WT (Fig. S7A, Supporting information), and the number of pregnancies and the number of pups per pregnancy in 12‐month‐old WT were also increased (Fig. S7B,C, Supporting information).

## Discussion

The deletion of *Nrg1* in granulosa cells of periovulatory follicles does not alter ovulation, cumulus cell–oocyte complex (COC) expansion or oocyte meiotic progression to the metaphase II (MII) stage; however, the matured oocytes in the mutant mice have a low cytostatic activity to arrest at the MII stage (Kawashima *et al*., 2014). After ovulation, spontaneous re‐resumption of meiosis is induced prematurely within 4 h resulting in a low pregnancy rate in gc*Nrg1*KO female mice (Kawashima *et al*., 2014). The recurrent succession of estrous cycles related to the low pregnancy rate appears to create changes in ovarian and pituitary functions that accelerate the accumulation of endocrine cells and the expansion of fibrous tissue in the stroma of gc*Nrg1*KO mice compared to events observed in the ovarian stroma of the WT mice.

Specifically, the mutant mice exhibit premature signs of aging that are eventually observed in older WT mice, including prolonged intervals between estrus; a reduced response to FSH; elevated serum levels of LH, FSH, and estradiol; and reduced levels of inhibin B. These changes except for the serum level of estradiol are often observed in women with increasing age (Santoro *et al*., 1996; Broekmans *et al*., 2007). In postmenopausal women, the level of estradiol was dramatically lower than that in younger women with reduce levels of inhibin B (Lee *et al*., 1988). However, it has been reported that the level of estradiol is increased during the specific period before menopause, and the level of estradiol was significantly higher in women that exhibit a reduced response to FSH than in normal women among middle‐aged women (from 38 to 42 years old) (Buyalos *et al*., 1997). Additionally, low responder patients, who exhibit a reduced response to exogenous FSH, also have fewer matured oocytes and the quality of the oocytes was poor compared to that observed in normal women (Jenkins *et al*., 1991). In this study, the number of oocytes fertilized and capable of developing to the blastocyst stage was significantly lower in 6‐month‐old gc*Nrg1*KO female mice as compared in those in the same age of WT mice. Thus, the mutant mice appear to provide a model to help explain not only the mechanisms leading to the decline in fertility with increasing age but also the reduced response of women to FSH with increasing age.

In the mutant mice, the endocrine and fertility changes that occur with increasing age were associated with the accumulation of aberrant endocrine cells in the ovarian stroma and the high expression of *Cyp17a1*,* Cyp19a1,* and *Lhcgr* in the stromal endocrine cells. The accumulation and function of these cells appeared to be driven, in part, by elevated LH. For example, the accumulation of endocrine cells in the ovarian stroma has previously been observed in ovaries of LH beta (LHβ) over‐expressing mice with increasing age (Risma *et al*., 1995). The importance of these stromal cells, as well as the gonadotropins, to the aging phenotype is also revealed by prolonged treatment of the aging mutant (6‐month‐old) and WT (12‐month‐old) mice with a GnRH antagonist. Notably, the antagonist treatment caused apoptosis and reduced expression of steroidogenic genes in the aberrant stromal endocrine cells and reduced the area of fibrosis tissue. The importance of the endocrine cells within the stroma to the aging phenotype is shown by the transplantation experiments, indicating that these endocrine cells in the aging ovaries have over‐ridden and superseded the ability of growing follicles to regulate the ovarian–pituitary–hypothalamic axis, such that high LH and FSH maintain elevated estradiol and testosterone production by these stromal cells.

The fibrosis in the ovarian stroma and increased levels of basal LH are also observed in women at advanced age (Gredmark *et al*., 1995; Matt *et al*., 1998) and in the patients with hypersecretion of LH (Schildkraut *et al*., 1996). In addition, LH‐induced androgen may be related to fibrosis within the ovary (Barbieri *et al*., 1986; Futterweit & Deligdisch, 1986). Thompson & Adelson (1993) reported that the androgen production occurred in ovarian stromal cells and increased with advanced age in women (Thompson & Adelson, 1993). Furthermore, ovarian stromal fibrosis was observed frequently in ovaries of hyperandrogenic patients, and the main source of androgen occurred in the stromal tissue (McNatty *et al*., 1980). In rodents, fibrosis is observed with increasing age (Briley *et al*., 2016) and treatment with exogenous testosterone induced fibrosis in the ovarian stroma (Miao *et al*., 2008). Our studies show that the stromal fibrosis also occurs in the gc*Nrg1*KO mice after 6 months of age and in WT mice at 12 months of age when both LH and androgens are chronically high and that this condition was reversed by prolonged treatment of GnRH antagonist. Collectively, the data in mice and women suggest that the fibrosis of ovarian stroma is dependent on LH‐androgen signaling and may be commonly induced in ovaries with increasing age.

However, the relationship between the growth of fibrosis tissue in the ovarian stroma and the suppression of follicle growth in the aging ovary has remained unclear. Follicular development is initiated by primordial follicle activation (Fortune *et al*., 2000) followed by progression to the secondary follicles stage that is dependent, in part, on oocyte‐secreted factors, such as GDF9 and BMP15 (Dong *et al*., 1996; Galloway *et al*., 2000). Further follicular development from secondary follicles is dependent on gonadotrophins, especially FSH (Dierich *et al*., 1998; Abel *et al*., 2000; Pakarainen *et al*., 2005). In the present study, increased fibrosis within the ovarian stroma was associated with the increased numbers of bilayer of secondary follicles, indicating that the fibrosis might suppress pre‐antral follicular development (Wood *et al*., 2015). Importantly, the long‐term treatment of GnRH antagonist not only increased the number of multilayer secondary follicles, but also reduced the fibrosis and the presence of aberrant endocrine cells in the stroma of the mutant mice. Thus, LH‐induced fibrosis in the ovarian stroma appears to alter the follicular‐stroma microenvironment to alter secondary follicle growth. Recently, Kawamura *et al*. (2013) reported that the growth of secondary follicles to the multilayer stage in ovaries of low FSH‐responding women was induced by the physical stimulation (cutting ovary) *in vitro* (Kawamura *et al*., 2013). They successfully promoted follicle growth and performed *in vitro* fertilization, and a healthy baby was delivered (Kawamura *et al*., 2013). The mechanisms by which mechanical stress appear to suppress follicle growth were explained by YAP/TAZ mechanotransduction system in granulosa cells (Kawamura *et al*., 2013). Information that an actin polymerization‐enhancing drug can induce follicular development would support the above idea (Cheng *et al*., 2015); however, in the present study, the strong intensity of F‐actin was only observed in the ovarian stroma and not within follicles. However, there are no reports yet relating the roles of mechanotransduction and the actin polymerization‐YAP/TAZ pathway during follicular development process in ovary using the knockout mice model. Therefore, further studies are required to determine the relationship among the abnormal endocrine condition, the growth of fibrosis tissue in ovarian stroma, and the suppression of follicular development in pre‐antral follicles. However, our studies indicate the restoration of fertility, the decrease in fibrosis, and growth of secondary follicles in ovarian stroma of aged ovary can be achieved by resetting serum levels of LH.

In conclusion, the appearance and maintenance of the aberrant endocrine cells and fibrous tissue within the ovarian stroma are dependent on chronic high levels of LH. The accumulation of these cells and tissue in the ovarian stroma appears to impact surrounding secondary follicles leading to impaired follicle development by an FSH‐independent manner. That long‐term treatment of GnRH antagonist normalized the endocrine functions and matrix conditions of the ovary and improved the fertility documents that the ovarian defects are LH‐dependent. Older women who respond poorly to exogenous FSH treatment may, like the aged gc*Nrg1*KO mice, have impaired follicular development associated with reduced levels of AMH, increased fibrosis of ovarian stroma caused by a high LH/high androgen condition, and hence reduced fertility. By reducing LH and removing the abnormal endocrine cells and fibrotic stromal tissue, it appears possible to reset the ovarian–hypothalamic–pituitary axis and ovarian functions and provides a novel strategy for improving fertility in a subset of aging women.

## Experimental procedures

### Materials

Pregnant mare serum gonadotropin (PMSG/eCG) and hCG were purchased from Asuka Seiyaku (Tokyo, Japan). Ganilelix acetate (SML0241), GnRH antagonist, was obtained from Sigma‐Aldrich (St. Louis, MO, USA). AMV reverse transcriptase from Promega (Madison, WI, USA), routine chemicals, and reagents were obtained from Sigma‐Aldrich or Nakarai Chemical Co (Osaka, Japan).

### Animals

Conditional depletion of *Nrg1* in ovarian granulosa cells (Kawashima *et al*., 2014) was achieved by crossing *Cyp19‐Cre* mice (Fan *et al*., 2008) with *Nrg1* ^*flox/flox*^ mice (Yang *et al*., 2001). The mutant mouse strains are in the C57BL/6 background.

Animals were housed in the Experiment Animal Center at Hiroshima University under a 14‐h light, 10‐h dark schedule and provided with food and water ad libitum. Animals were treated in accordance with the NIH Guide for the Care and Use of Laboratory Animals, as approved by the Animal Care and Use Committee at Hiroshima University.

### Mating test

The mating experiment was conducted using five females of each genotype (*Nrg1* ^*flox/flox*^, WT and *Nrg1* ^*flox/flox*^ *;Cyp19a1‐Cre*, gc*Nrg1*KO) that were 2 months old at the outset. Adult WT male mice were placed in each cage for 15 months, and the number of pups in each litter and the days of pregnancy were recorded. The dispersion of interval between pregnancies was compared by Bartlett's test.

### Vaginal smear analysis

Vaginal smear analyses were carried out according to Rascop *et al*. (1966). Smears were obtained daily between 09:00 and 10:00 am. The fire‐polished, shortened tip of a Pasteur pipet was placed at the vagina.

### Morphological classification of follicles

The classification of follicles was performed according to Britt *et al*. (2000). Paraffin‐embedded ovarian sections taken at intervals of 30 μm were mounted on slides. Routine hematoxylin and picrosirius red staining were performed for histologic examination by light microscopy. Follicle numbers in 12 sections per ovary were evaluated as primordial follicles (oocytes surrounded by a single layer of flatness granulosa cells), primary follicles (oocyte surrounded by a single layer of cuboidal granulosa cells), pre‐antral follicles (oocyte surrounded by two or more layers of granulosa cells with no antrum), antral follicles (antrum within the granulosa cell layers enclosing the oocyte), corpus luteum (the occupation of lutein cells in follicle surrounded by basal lamina), or corpus albicans (basal lamina collapsed and lutein cells hyalinized). Follicles were determined to be atretic if they displayed two or more of the following criteria within a single cross section: more than two pyknotic nuclei, granulosa cells within the antral cavity, granulosa cells pulling away from the basement membrane, or uneven granulosa cell layers.

### RNA extraction and quantitative RT–PCR analyses

Total RNA was obtained from whole ovaries using the RNAeasy Mini Kit (Qiagen Sciences, Germantown, MD, USA) according to the manufacturer's instructions. Total RNA was reverse transcribed using 500 ng poly‐dT and 0.25 U avian myeloblastosis virus reverse transcriptase at 42 °C for 75 min and 95 °C for 5 min. Quantitative real‐time PCR analyses were performed as previously (Shimada *et al*., 2008). Briefly, cDNA and primers shown in Table S1 (Supporting information) were added to 15 μL total reaction volume of the Power SYBR Green PCR Master Mix (Applied Biosystems, Foster City, CA, USA). PCRs were then performed using the StepOne real‐time PCR system (Applied Biosystems). Conditions were set to the following parameters: 10 min at 95 °C followed by 45 cycles each of 15 s at 95 °C and 1 min at 64 °C. *L19* was used as a control for reaction efficiency and variations in concentrations of mRNA in the original RT reaction.

### Immunohistochemistry

Ovaries were fixed in 4% paraformaldehyde overnight, dehydrated in 70% (v/v) ethanol, and embedded in paraffin. The paraffin‐embedded fixed sections (4 μm) were deparaffinized in xylene washes and quenched with 3% hydrogen peroxide in methanol. The sections were incubated with 20% (v/v) nonimmune goat serum/PBS to block nonspecific sites followed by incubation with primary antibody overnight at 4 °C (1:100 of anti‐LH receptor antibody (catalog # bs‐0984); Bioss, Woburn, MA, USA; 1:100 of anti‐CYP19a1 antibody (catalog # BS2516); Bioworld Technologies, St Louis Park, MN, USA; 1:300 of anti‐CYP17a1 antibody (catalog # bs‐3853); Bioss). The positive signals were visualized using VECTASTAINE life ABC rabbit IgG kit (Vector Laboratories, Burlingame, CA, USA) according to the manufacturer's recommendations. The area of anti‐LH receptor antibody‐positive cells in ovarian stroma was analyzed using BZ‐II application software (Keyence, Tokyo, Japan).

### The detection of actin polymerization by rhodamine phalloidin staining

Ovaries were collected and fixed in 4% paraformaldehyde, embedded in paraffin, and processed by routine procedures as described above. Sections were stained with rhodamine phalloidin (1:100, Invitrogen, Carlsbad, CA, USA) at 37 °C for 60 min and then were further stained with 4′,6‐diamidino‐2‐phenylindole (DAPI) (VECTASHIELD Mounting Medium with DAPI; Vector Laboratories). Digital images were captured using a BZ‐9000 microscope (Keyence).

### The collection of ovulated COCs and *in vitro* fertilization

The mice were injected intraperitoneally (ip) with 4 IU of eCG (Asuka Seiyaku) to stimulate pre‐ovulatory follicle development followed 48 h later with 5 IU of hCG (Asuka Seiyaku) to stimulate ovulation. *In vitro* fertilization was analyzed as described previously (Shimada *et al*., 2008). COCs that were collected from oviduct at 16 h after hCG were placed in 50 μL of human tubal fluid (HTF) medium. Spermatozoa were collected from the cauda epididymis of each genotype mice into 500 μL of HTF medium. After 60‐min incubation to induce sperm capacitation, the spermatozoa were introduced into the fertilization medium at a final concentration of 1 000 spermatozoa/μL. Twelve hours after insemination, all gametes were further cultured for an additional day in the developing medium (KSOM + AA, Millipore, Billerica, MA, USA) to check for development to the blastocyst stage.

### Ovarian replacement

Ovarian replacement was performed according to Pakarainen *et al*. (2005). Briefly, the mice were anesthetized with 100 μL of 20% pentobarbital. Both ovaries were removed surgically from the bursa surrounding ovaries and each was replaced with ovaries from another donor mouse, and skin was sutured after the operation.

### Long‐term treatment of GnRH antagonist

Injections of 50 μg/kg BW of GnRH antagonist (Sigma‐Aldrich) were given daily at 09:00 am. With the injection of GnRH antagonist, smear analysis was carried out and the injection was stopped when diestrus periods was observed. The mice after injection were mated with 3‐month‐old WT male mice for 3 months, and the number of pups and deliver was record.

### Measurement of AMH, FSH, LH, testosterone, inhibin B, estradiol, and progesterone

Serum levels of AMH were measured using mouse AMH ELISA kit (Elabscience Biotechnology, Bethesda, MD, USA); serum FSH was measured using the FSH ELISA kit (Abnova, Taipei, Taiwan); and serum LH, testosterone, estradiol, and progesterone were measured using specific ELISA kits (Endocrine Technology, Inc, Newark, CA, USA). Inhibin B was measured using the mouse Inhibin B Enzyme Immunoassay Kit (RayBiotech, Norcross, GA, USA). All measurements were taken according to the manufacturer's instructions.

### Statistics

Statistical analyses of data from three or four replicates for comparison were carried out by either Student's *t*‐test or one‐way ANOVA followed by Student's *t*‐test (Statview; Abacus Concepts, Inc., Berkeley, CA, USA). In Figure 3 and Figure 4, the data obtained from three different cultures were carried out by a two‐way ANOVA (Statview; Abacus Concepts, Inc).

## Author contributions

TU was responsible for experimental design, data analysis, and wrote the manuscript. TK, IK, KT, SO, and HK collected samples and interpreted data. JSR was responsible for planning and discussions, and reviewed the manuscript. MS supervised all aspects of this work and wrote the manuscript.

## Funding

This work was supported in part by The Japan Society for the Promotion of Science (JSPS) KAKENHI, JP24688028, JP 16H05017 (to MS) and JP15J05331 (to TU), by Japan Agency for Medical Research and Development (AMED) 16gk0110015 h0001 (to MS), and by National Institute of Health (NIH)‐HD‐076980 (to JSR).

## Conflict of interest

The authors have nothing to disclose.

## Supporting information


**Fig. S1** The patterns of estrous cycles were determined by vaginal smear analysis for 15 days.
**Fig. S2** Serum levels of FSH, LH, estradiol, testosterone, and inhibin B in WT and gc*Nrg1*KO.
**Fig. S3** (A) Low magnification images stained using rhodamine phalloidin and DAPI with constant settings that were established for the 12‐month old gc*Nrg1*KO. (B) An image of the ovary in 12‐month‐old gc*Nrg1*KO mice stained without rhodamine phalloidin. Scale bars correspond to 300 μm.
**Fig. S4** The number of follicles at each stage of development per mm^2^ in ovaries of WT and gc*Nrg1*KO mice at each age (*n* = 3 ovaries per each group).
**Fig. S5** (A) The expression of CYP19, CYP17 and LHR in ovaries 48 h after eCG treatment. (B) Image of an ovary in a 6‐month‐old gc*Nrg1*KO mouse stained without primary antibody.
**Fig. S6** (A) The appearance of cleaved caspase 3‐positive cells, LHR‐positive cells or CYP19‐positive cells in the ovarian stroma. (B) The percent of each stage of secondary follicle (assessed by layers) in ovaries of 6‐month‐old WT, 6‐month‐old gcNrg1KO, and 6‐month‐old gcNrg1KO mice treated with the GnRH‐antagonist.
**Fig. S7** (A) Estrous cycle patterns analyzed for 13 days in 12‐month‐old WT mice injected with GnRH‐antagonist (D, diestrus P, Proestrus E, Estrus WE, Weak estrus M, Metestrus). (B) The number of pups per delivery was calculated in 12‐month‐old WT treated with saline (12 mon WT Cont) or GnRH‐antagonist (12 mon WT Anta). (C) The number of pups delivered during 3 months was calculated in 12‐month‐old WT mice treated with saline (12 mon WT Cont) or GnRH‐antagonist (12 mon WT Anta).

**Table S1** List of primers employed for RT‐PCR and the expected size.
