# Supplementary material for: The acceleration of reproductive aging in Nrg1 flox/flox ;Cyp19‐Cre female mice
Source: Aging Cell. 2017 Aug 31;16(6):1288–99. doi: 10.1111/acel.12662 (PMC5676068; doi:10.1111/acel.12662)
Supplement: Supplementary file 1 — Fig. S1 The patterns of estrous cycles were determined by vaginal smear analysis for 15 days. Fig. S2 Serum levels of FSH, LH, estradiol, testosterone, and inhibin B in WT and gcNrg1KO. Fig. S3 (A) Low magnification images stained using rhodamine phalloidin and DAPI with constant settings that were established for the 12‐month old gcNrg1KO. (B) An image of the ovary in 12‐month‐old gcNrg1KO mice stained without rhodamine phalloidin. Scale bars correspond to 300 μm. Fig. S4 The number of follicles at each stage of development per mm2 in ovaries of WT and gcNrg1KO mice at each age (n = 3 ovaries per each group). Fig. S5 (A) The expression of CYP19, CYP17 and LHR in ovaries 48 h after eCG treatment. (B) Image of an ovary in a 6‐month‐old gcNrg1KO mouse stained without primary antibody. Fig. S6 (A) The appearance of cleaved caspase 3‐positive cells, LHR‐positive cells or CYP19‐positive cells in the ovarian stroma. (B) The percent of each stage of secondary follicle (assessed by layers) in ovaries of 6‐month‐old WT, 6‐month‐old gcNrg1KO, and 6‐month‐old gcNrg1KO mice treated with the GnRH‐antagonist. Fig. S7 (A) Estrous cycle patterns analyzed for 13 days in 12‐month‐old WT mice injected with GnRH‐antagonist (D, diestrus P, Proestrus E, Estrus WE, Weak estrus M, Metestrus). (B) The number of pups per delivery was calculated in 12‐month‐old WT treated with saline (12 mon WT Cont) or GnRH‐antagonist (12 mon WT Anta). (C) The number of pups delivered during 3 months was calculated in 12‐month‐old WT mice treated with saline (12 mon WT Cont) or GnRH‐antagonist (12 mon WT Anta). [file ACEL-16-1288-s001.pdf]

Supplemental Figure 1.
The patterns of estrous cycles were determined by vaginal smear analysis for 15 days. (D, diestrus P, Proestrus E, Estrus WE, Weak estrus M, Metestrus)

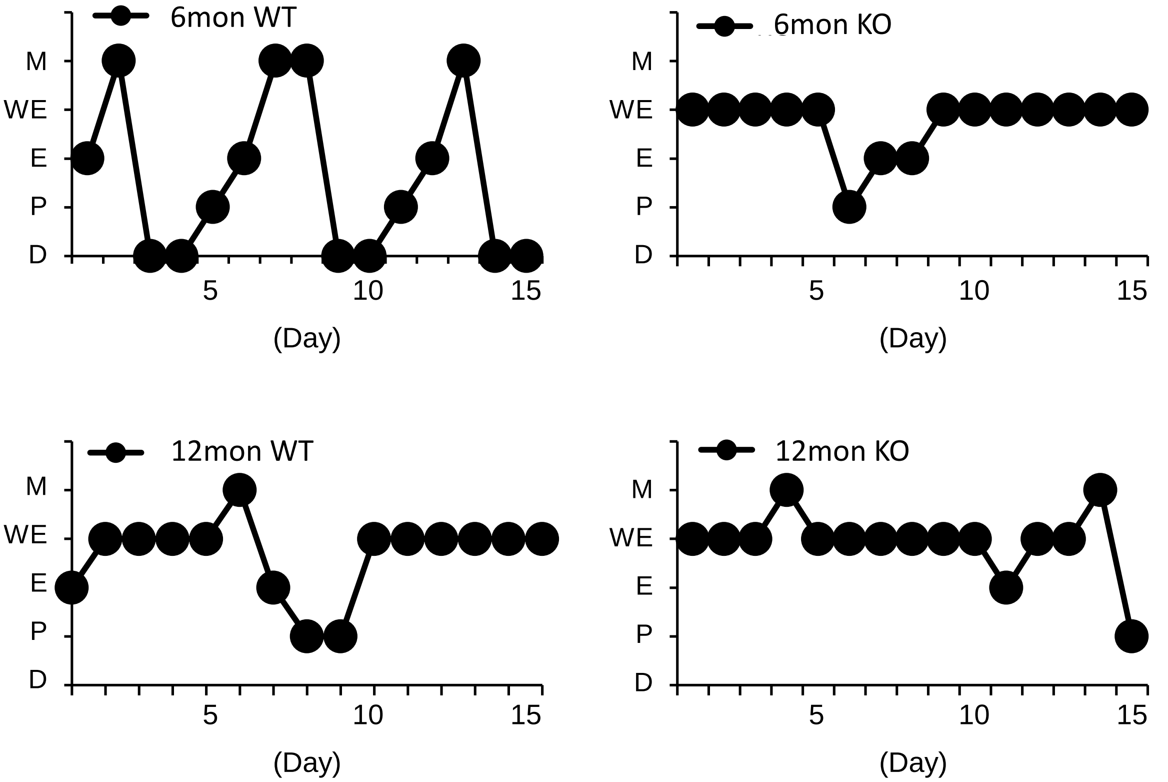

Supplemental Figure 1

Supplemental Figure 2. Serum levels of FSH, LH, estradiol, testosterone, and inhibin B in WT and gc*Nrg1*KO.
The samples from 6-month-old mice were collected for 4 days. The levels of each hormone were measured by EIA kit. N=4 animals for each genotype. Values are represented as the mean +/-SEM of four replicates. Different superscripts denote significant differences among the estrus cycle in each genotype ($p<0.05$).

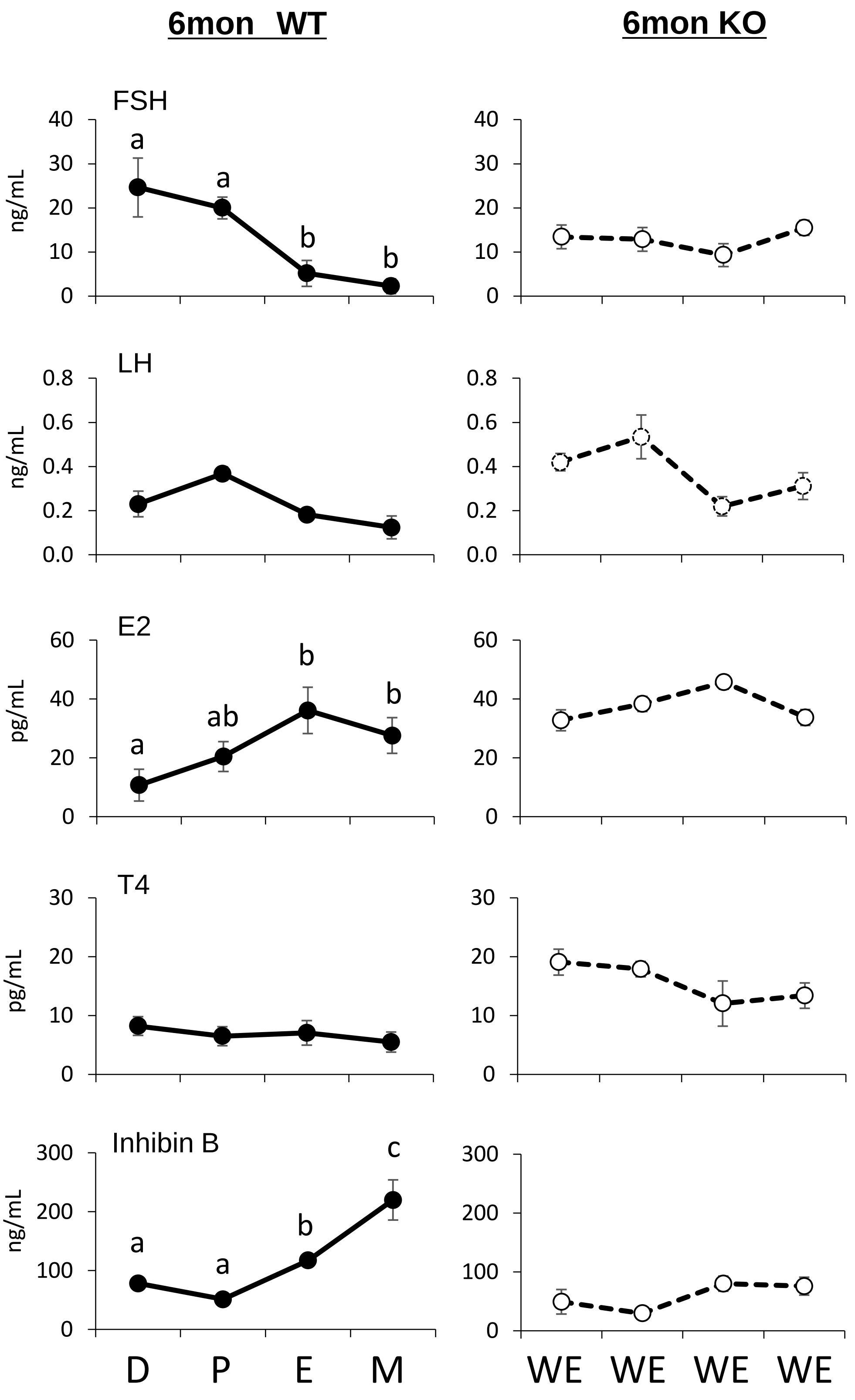

Supplemental Figure 2

Supplemental Figure 3.
(A) Low magnification images stained using rhodamine phalloidin and DAPI with constant settings that were established for the 12-month old gc*Nrg1*KO. Scale bars in image are 300 µm.
(B) An image of the ovary in 12-month-old gc*Nrg1*KO mice stained without rhodamine phalloidin. Scale bars correspond to 300 µm.

(A)

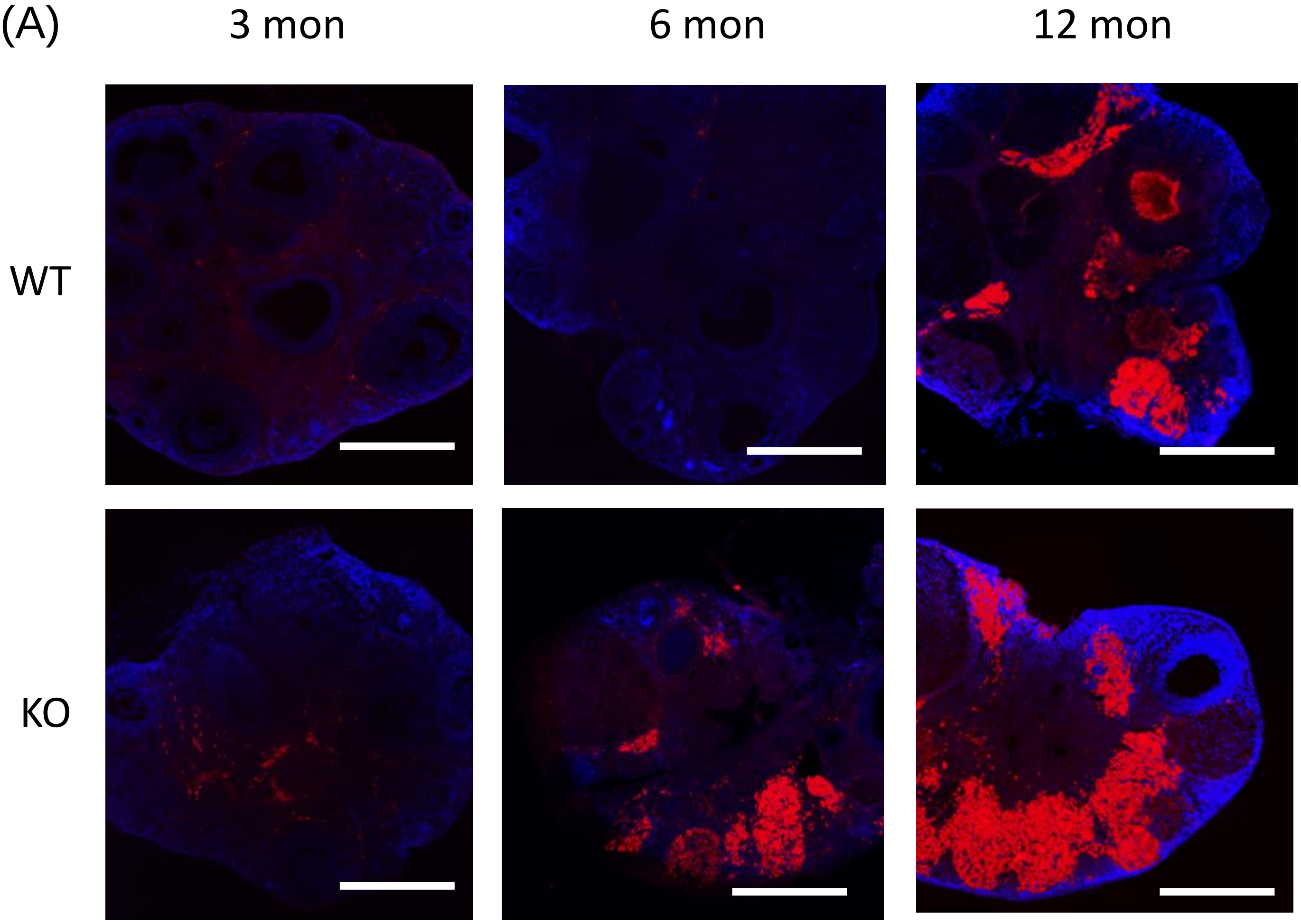

(B)

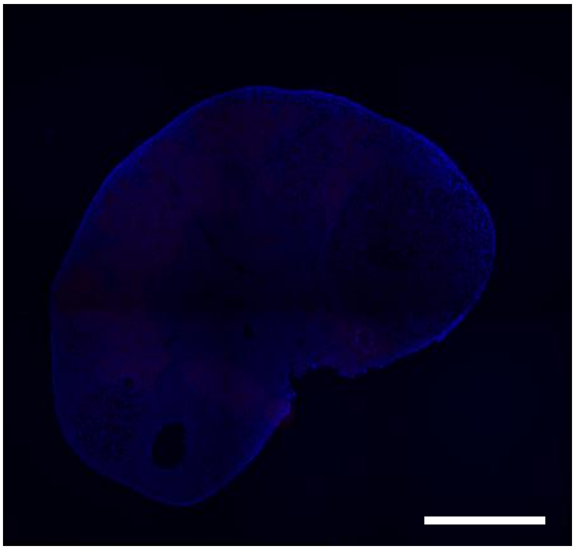

Supplemental Figure 3

Supplemental Figure 4.

The number of follicles at each stage of development per mm^2 in ovaries of WT and gc*Nrg1*KO mice at each age (n=3 ovaries per each group). Sections taken at intervals of 30 µm, and 6 µm paraffin-embedded ovaries were mounted on slide. Follicle numbers in 12 sections per ovary were evaluated. Values are represented as the mean +/-SEM of three replicates. *denotes the significant differences observed between genotypes at the same age ($p<0.05$). Different superscripts denote significant differences among the age in each genotype ($p<0.05$). (Pm, Primordial follicle Pr, Primary follicle Sec, Secondary follicle Ant, Antral follicle CL, Corpus luteum CA, Corpus albicans Atr, Atretic follicle)

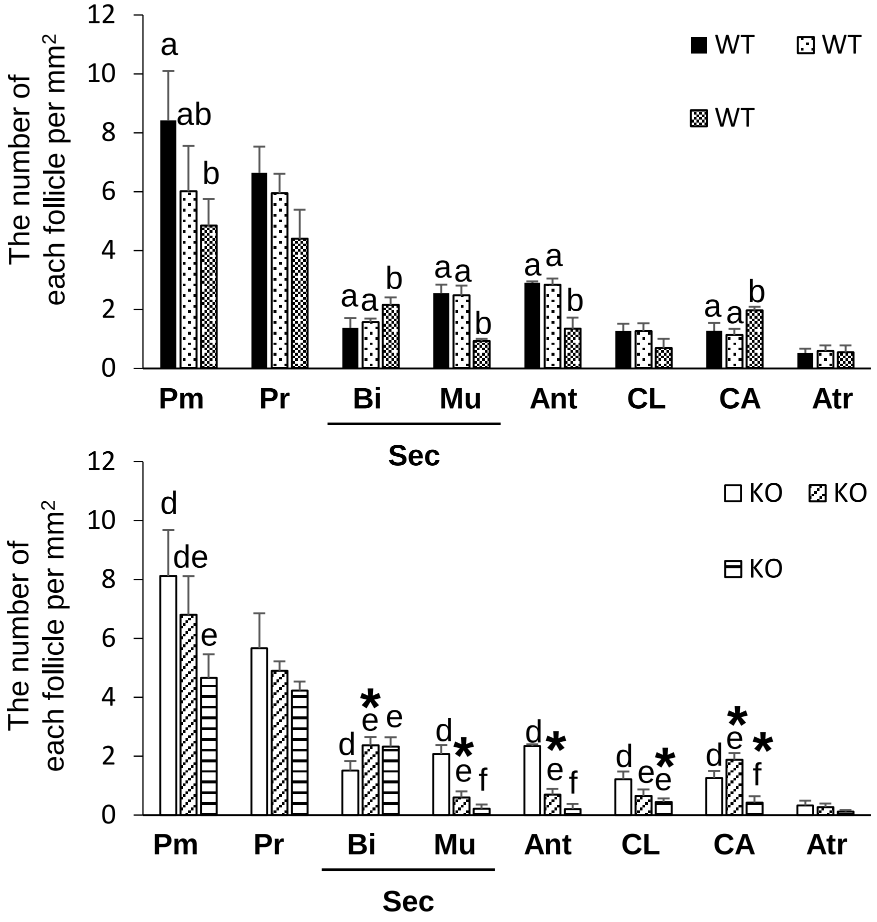

Supplemental Figure 4

Supplemental Figure 5.
(A) The expression of CYP19, CYP17 and LHR in ovaries 48 hours after eCG treatment. Scale bars correspond to 300 µm in low magnification (×100) and 100 µm in high magnification (×400).
(B) Image of an ovary in a 6-month-old gcNrg1KO mouse stained without primary antibody. Scale bars correspond to 300 µm in low magnification (×100) and 100 µm in high magnification (×400).

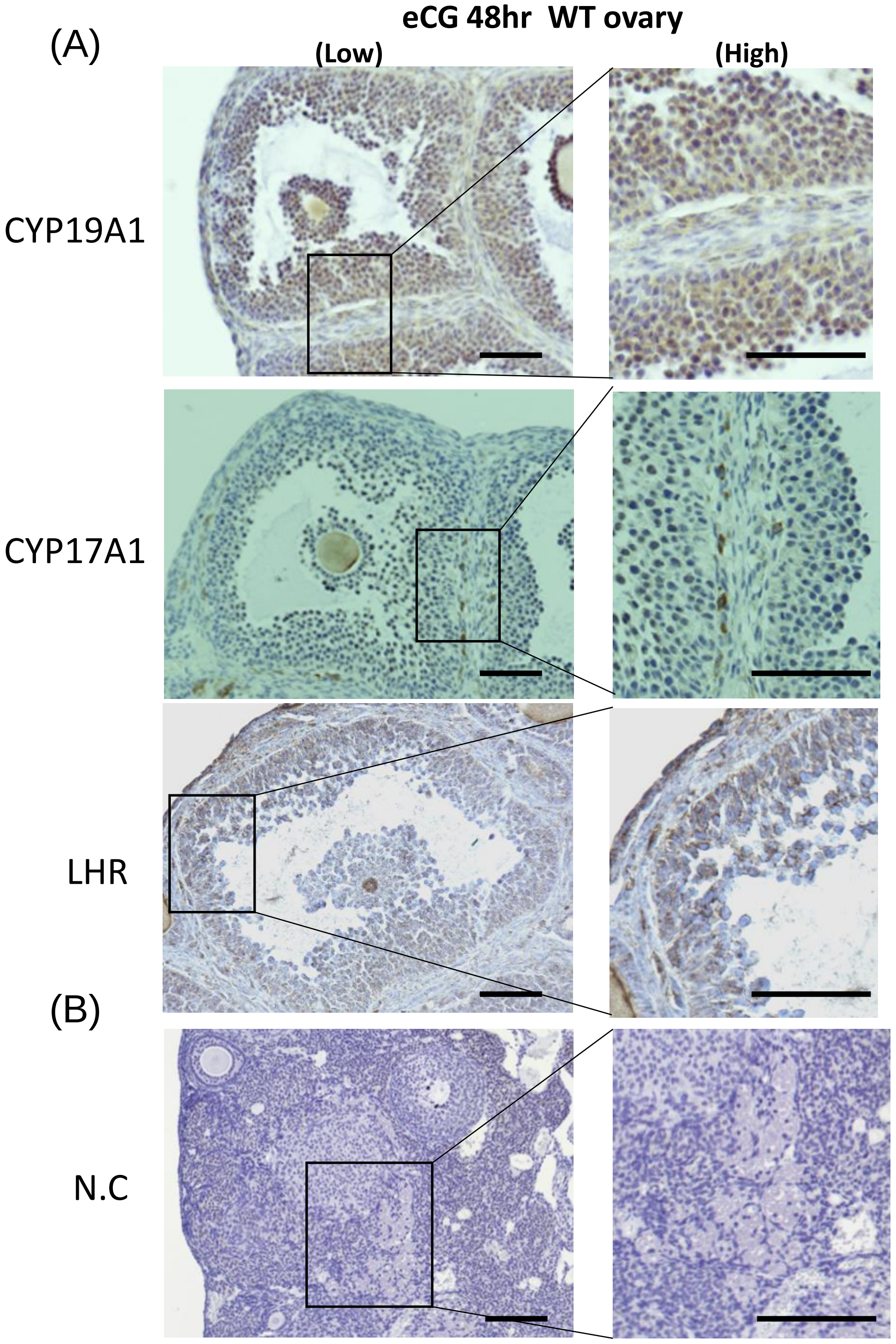

Supplemental Figure 5

Supplemental Figure 6.
(A) The appearance of cleaved caspase 3-positive cells, LHR-positive cells or CYP19-positive cells in the ovarian stroma. The ovaries were collected before treatment (Day 0) and day 6 after GnRH antagonist injection. Scale bar corresponded 200 µm.
(B) The percent of each stage of secondary follicle (assessed by layers) in ovaries of 6-month-old WT, 6-month-old gcNrg1KO, and 6-month-old gcNrg1KO mice treated with the GnRH-antagonist. Sections taken at intervals of 30 µm, and 6 µm paraffin-embedded ovaries were mounted on slide. Follicle numbers in 12 sections per ovary were evaluated. Values are represented as the mean +/-SEM of three replicates. *, $P < .05$, significant differences were observed.

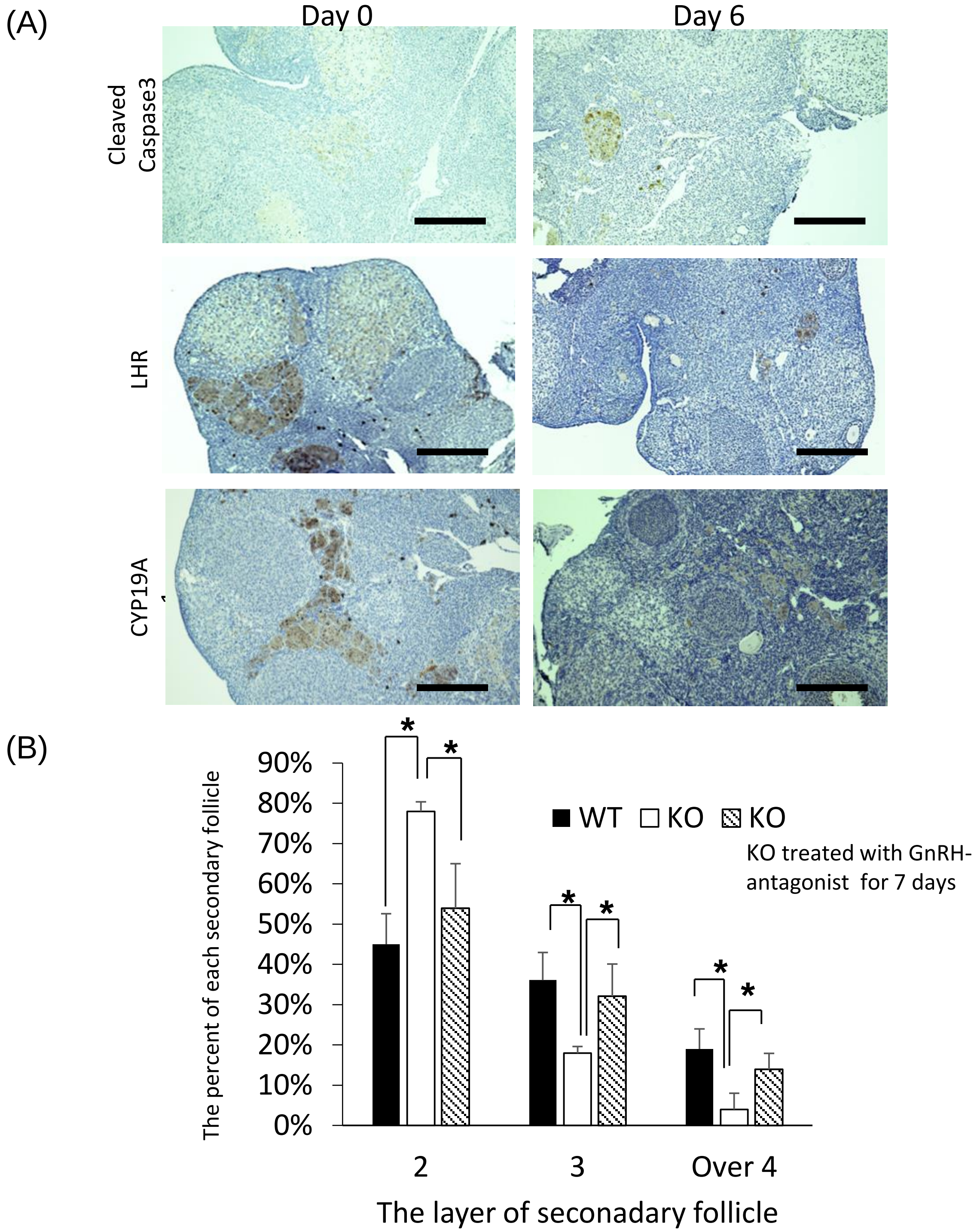

Supplemental Figure 6

Supplemental Figure 7.

(A) Estrous cycle patterns analyzed for 13 days in 12-month-old WT mice injected with GnRH-antagonist (D, diestrus P, Proestrus E, Estrus WE, Weak estrus M, Metestrus). When estrous cycles reached diestrus, the treatment of GnRH-antagonist was stopped. Five mice injected with GnRH-antagonist were checked their estrous cycles. Each colored line represents the estrous cycles of each mouse.

(B) The number of pups per delivery was calculated in 12-month-old WT treated with saline (12 mon WT Cont) or GnRH-antagonist (12 mon WT Anta). Three pairs were prepared in each treatment. *denotes a significant difference ($p<0.05$).

(C) The number of pups delivered during 3 months was calculated in 12-month-old WT mice treated with saline (12 mon WT Cont) or GnRH-antagonist (12 mon WT Anta). Five pairs were prepared in each treatment. *denotes a significant difference ($p<0.05$).

(A)

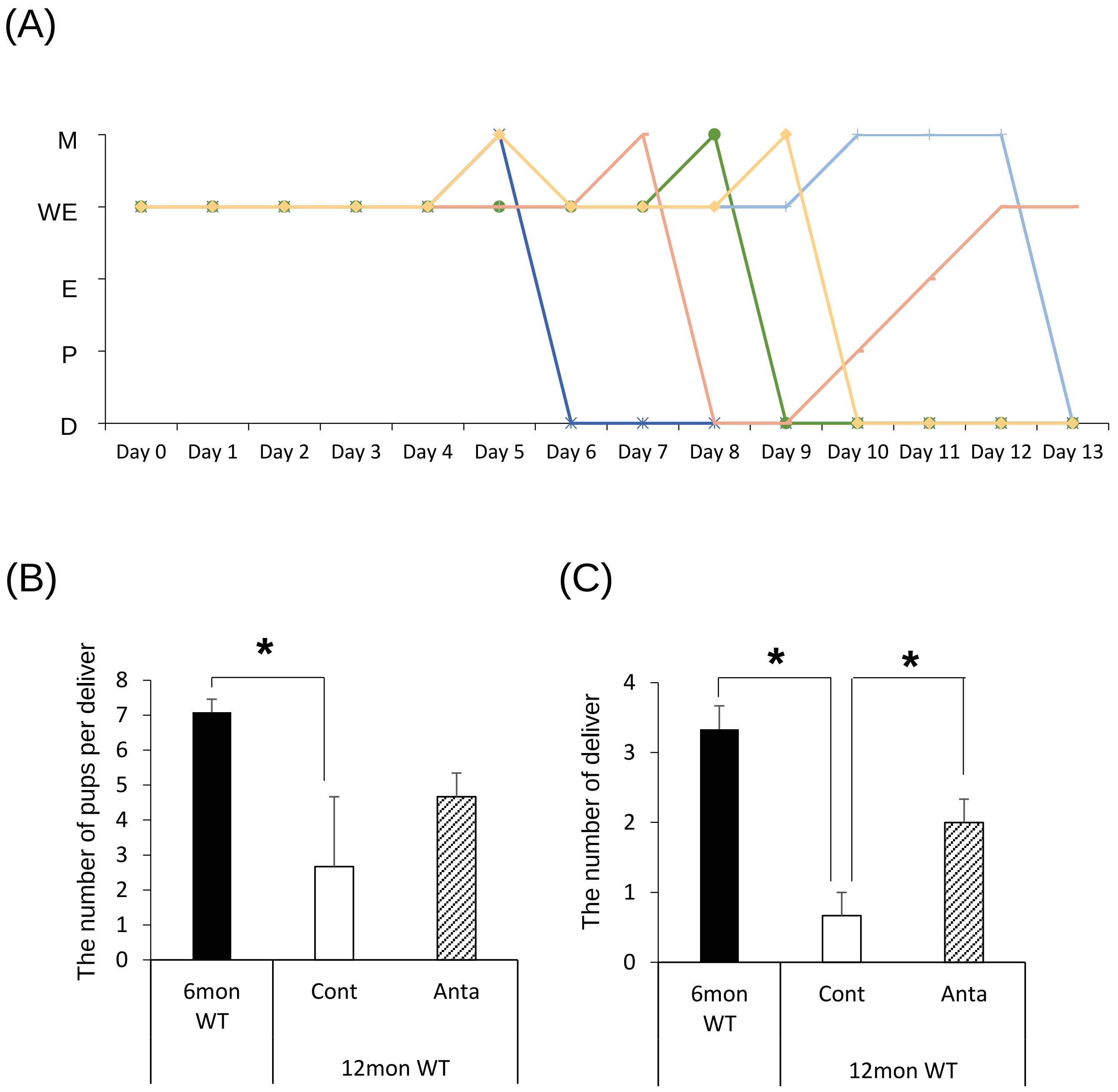

Supplemental Figure 7
