# Supplementary material for: The acceleration of reproductive aging in Nrg1 flox/flox ;Cyp19‐Cre female mice
Source: Aging Cell. 2017 Aug 31;16(6):1288–99. doi: 10.1111/acel.12662 (PMC5676068; doi:10.1111/acel.12662)
Supplement: Supplementary file 2 — Table S1 List of primers employed for RT‐PCR and the expected size. [file ACEL-16-1288-s002.docx]

Supplemental Table1. List of primers employed for RT-PCR and the expected size

| Gene | Forward Primer | Reverse Primer | Size | Anneling  temperature |
| --- | --- | --- | --- | --- |
| *L19* | 5’-GGCATAGGGAAGAGGAAGG-3’ | 5’-GGATGTGCTCCATGAGGATGC-3’ | 199 | 60 |
| *Col3a1* | 5’-AGCCACCTTGGTCAGTCCTA-3’ | 5’-GTGTAGAAGGCTGTGGGCAT-3’ | 184 | 60 |
| *Cyp11a1* | 5’-GGGAGACATGGCCAAGATGG-3’ | 5’-CAGCCAAAGCCCAAGTACCG-3’ | 279 | 60 |
| *Cyp17a1* | 5’-GGCCCCAGATGGTGACTCT-3’ | 5’-GGGACTCCCCGTCGTATGTA-3’ | 205 | 64 |
| *Cyp19a1* | 5’-TTGCACCCAAATGAGGACAG-3’ | 5’-CTTCACTGGTCCCCAACACA-3’ | 290 | 60 |
